# Mandibular Ossifying Fibroma and Multiple Oral Papillomas in a Roe Deer (*Capreolus capreolus*)

**DOI:** 10.3389/fvets.2020.00166

**Published:** 2020-04-02

**Authors:** Samoa Zürcher-Giovannini, Thomas-Daniel Ruder, Roy Pool, Karoly Erdelyi, Francesco C. Origgi

**Affiliations:** ^1^Centre for Fish and Wildlife Health, Vetsuisse Faculty, University of Bern, Bern, Switzerland; ^2^Centre of Forensic Imaging and Virtopsy, Institute of Forensic Medicine, University of Bern, Bern, Switzerland; ^3^Department of Forensic Medicine and Imaging, Institute of Forensic Medicine, University of Zurich, Zurich, Switzerland; ^4^Department of Pathobiology, College of Veterinary Medicine & Biomedical Sciences, Texas A&M University, College Station, TX, United States; ^5^Veterinary Diagnostic Directorate, National Food Chain Safety Office, Budapest, Hungary

**Keywords:** roe deer, ossifying fibroma, computed tomography, papilloma, wildlife

## Abstract

An emaciated, adult, free-ranging roe deer (*Capreolus capreolus*) presenting a large mandibular mass, was shot by a game warden in Sissach, Switzerland. The head of the roe deer was submitted to the Center for Fish and Wildlife Health for examination. Grossly, the mass consisted of a 6 × 7 × 4 cm mandibular exophytic growth, associated with loss of incisors teeth. On cut section, a hard, light-tan core was rimmed by a thick layer of soft tissue. Computed tomography examination confirmed the mandibular origin of the mass. Histologically, the mass consisted of an unencapsulated fibro-osseous neoplasm. The bony portion was composed of multiple haphazardly arranged spicules rimmed by osteoblasts with no associated periosteal layer. Embedding the bony spicules were short anastomosing and branching streams and bundles of spindled cells. The overlaying partially ulcerated mucosa, showed prominent rete ridges deepening into the submucosa. In addition to the mandibular mass, multiple soft cauliflower-like proliferations were expanding from the gingival surface. Histologically, these masses were arranged in papillary elements composed of pluristratified squamous epithelium with long rete ridges extending into a rich underlying fibrovascular supportive stroma. Neither papillomaviral DNA nor antigen could be identified in association with the oral masses. The gross, histological and radiological features of the mandibular mass are consistent with an ossifying fibroma, while the cauliflower oral masses were diagnosed as papillomas.

## Background

Ossifying fibroma is a benign fibro-osseous neoplasm, which most frequently develops within the mandible. This tumor is considered a rare occurrence in veterinary medicine and it has been reported mainly in young horses (1). Other species including cats (2), Anglo-Nubian goats (3), greater kudu (4), sheep (5), miniature Rex rabbits (6) and dogs (7) may also, but more rarely, develop the tumor.

Papillomas and fibropapillomas have been reported in a wide range of domestic and wild animals including several cervid species, such as white-tailed (*Odocoileus virginianus*) and mule deer (*Odocoileus hemionus*) in North America (8), European elk (*Alces alces*) (9) and reindeer (*Rangifer tarandus*) in Sweden (10), red deer (*Cervus elaphus*) in Scotland (11), as well as red deer and roe deer (*Capreolus capreolus*) in Hungary (12, 13). Species-specific papillomaviruses (PV), whose origin has been recently traced to the European continent (14), were frequently detected in association with these tumors. Fibropapillomas in roe deer most commonly develop on the skin of the head, neck, abdomen and limbs of the affected animal (15).

In this short communication, we describe the simultaneous occurrence of a mandibular ossifying fibroma and multiple oral papillomas in a wild ruminant. To the best of our knowledge, this is the first documented description of an ossifying fibroma in a roe deer (*Capreolus capreolus*). Additionally, the papillomas described in this study, developed within the oral cavity differently from the more common cutaneous papilloma and fibropapillomas reported in deer up to date.

## Case History

A weak and emaciated free-ranging adult (estimated age 4, 5 years old, according to the feature of the dentition), female roe deer (*Capreolus capreolus*) presenting a large mass expanding from the cranial aspect of the mandible, was culled by a game-warden in Sissach, Switzerland. A field necropsy was performed by the game warden who did not observe any additional gross abnormalities. The head of the roe deer was submitted to the Center for Fish and Wildlife Health (FIWI) at the University of Bern, Switzerland, for a full pathology investigation. During the examination of the submitted head, additionally to the mandibular mass, multiple proliferative growths were observed expanding from the upper and lower gingiva.

Samples of the masses were fixed in 10% buffered formalin, decalcified for about 1 h and then processed routinely for histological examination. Paraffin sections (5 μm thick) were stained with hematoxylin and eosin (H&E).

A computed tomography (CT) scan of the whole head was performed at the Institute of Forensic Medicine in Bern, Switzerland.

## Description of Laboratory Investigations and Diagnostic Tests

### Pathology and Imaging

**Figure 1:**
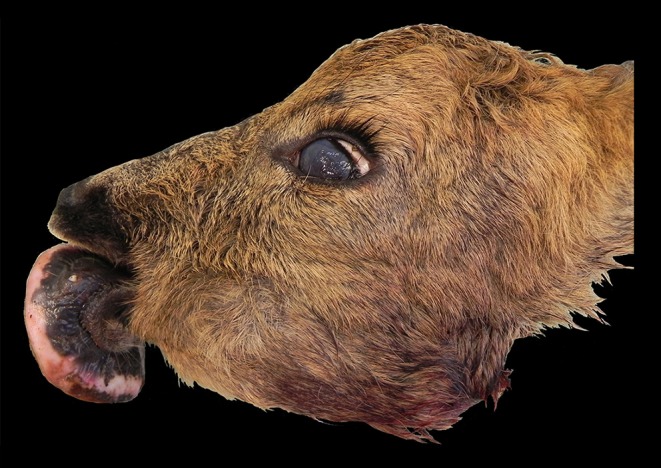
Head, roe deer (*Capreolus capreolus*); a mass is expanding from the rostral aspect of the mandible.

**Figure 2:**
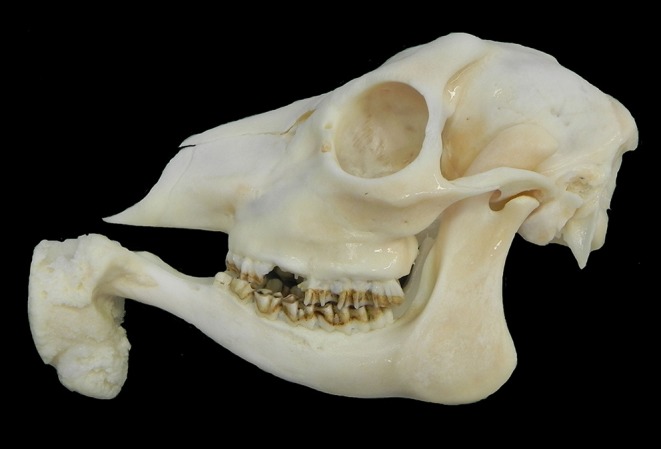
Skull, roe deer; the mass is composed of a bony center.

**Figure 3:**
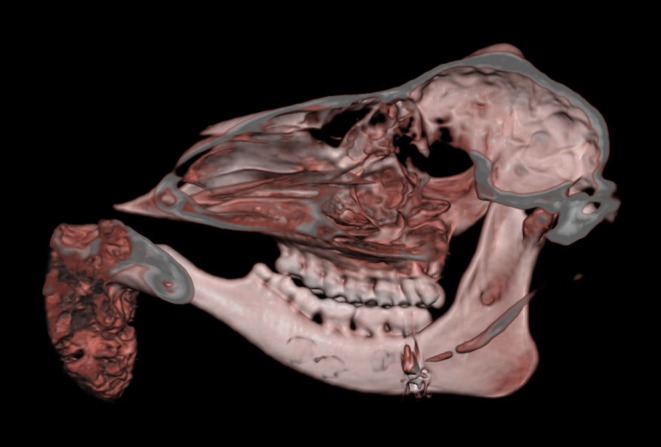
Head, roe deer; computed tomography scan showing the contiguity of the bony mass with the mandible and the effacement of the latter by the tumor. This figure uses *volume-rendering* technique to provide a three-dimensional image of the skull.

**Figure 4:**
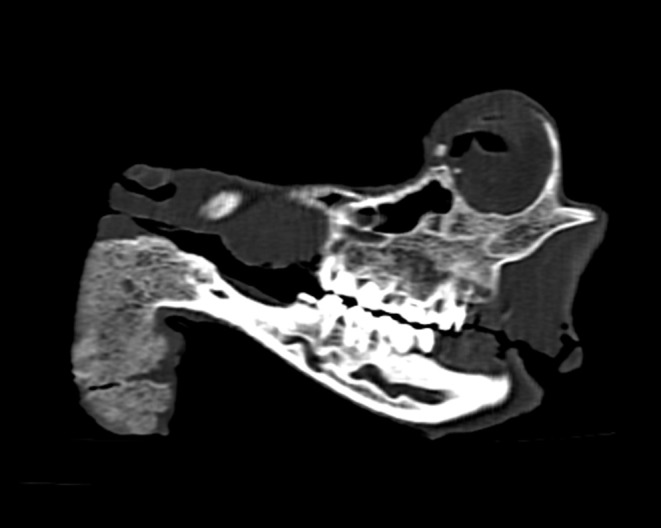
Head, roe deer; computed tomography scan showing the contiguity of the bony mass with the mandible and the effacement of the latter by the tumor. This figure features a single sagittal MPR (multi-planar reconstruction) image through the skull at the level of the tumor.

Macroscopically, the mandibular mass consisted of a 6 × 7 × 4 cm exophytic growth, expanding from the rostral aspect of the mandible and associated with loss of incisors teeth (Figures 1, 2). The mass was covered laterally and dorso-caudally by intact mucosa contiguous to that of the oral cavity, while a local-extensive mucosal ulceration was present on its rostral aspect. On cut section, the mass was composed of a hard, light-tan core rimmed by a thick layer of soft tissue. CT examination revealed the presence of both osseous and soft tissue components, and confirmed that the neoplasm was originating from the mandible (Figures 3, 4). The portion of the mandible contiguous to the mass showed local bone destruction. Both bodies of the mandible were unaffected.

**Figure 5:**
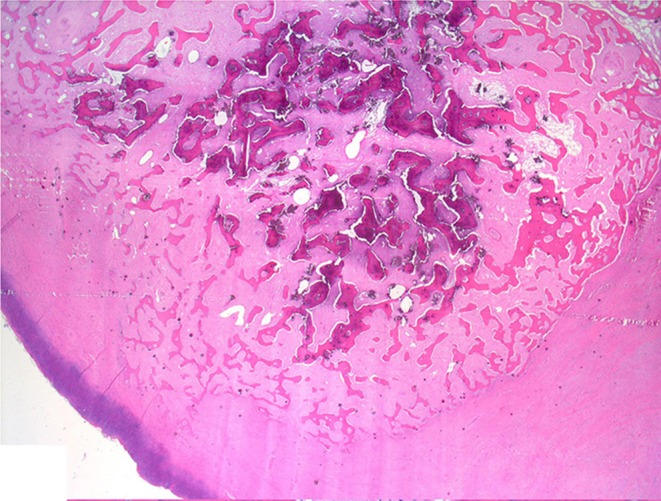
Mandibular mass, roe deer (*Capreolus capreolus*); a proliferative fibrous-osseous mass comprising cancellous bone in the absence of a periosteal layer is elevating the ulcerated labial mucosa; H&E stain.

**Figure 6:**
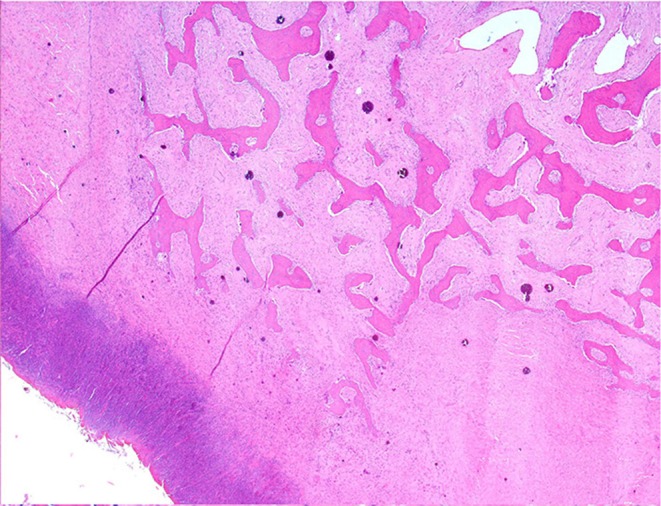
Mandibular mass, roe deer; the bony spicules increase in size from the periphery to the center of the mass; H&E stain.

**Figure 7:**
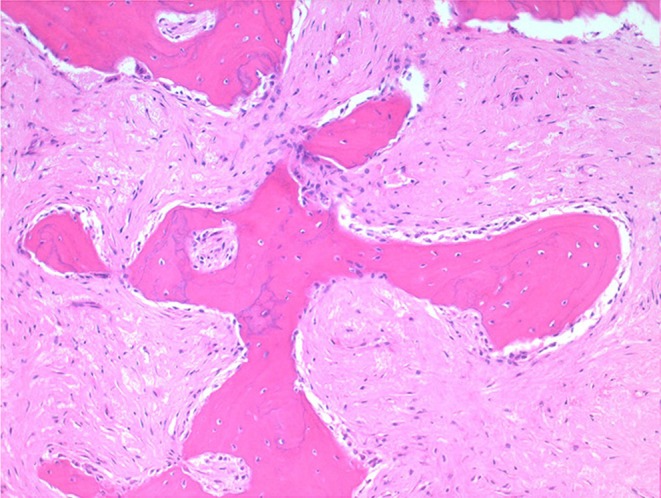
Mandibular mass, roe deer; embedding the bony spicules were streams and bundles of spindled cells supported by an abundant eosinophilic matrix; H&E stain.

**Figure 8:**
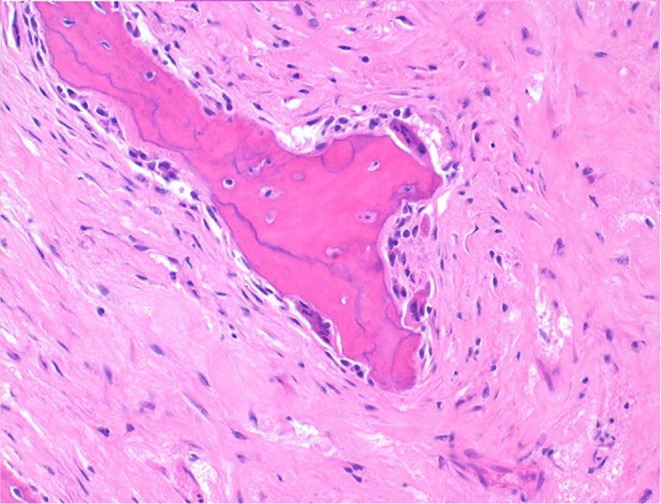
Mandibular mass, roe deer; spicules are lined by osteoblasts and those larger and deeper seated in the mass undergo bone remodeling at their surfaces; H&E stain.

Histologically, the mass consisted of an unencapsulated fibro-osseous neoplasm, expanding the oral submucosa and elevating the partially ulcerated mucosa (Figure 5). The bony portion was composed of multiple haphazardly arranged spicules of woven bone rimmed by polygonal cells (osteoblasts). This cancellous bone mass was not surrounded by a layer of periosteum, consistent with the edge of an expanding fibrous stromal tumor mass (Figure 6). Embedding the bony spicules were short anastomosing and branching streams and bundles of spindled cells supported by an abundant eosinophilic matrix (Figures 7, 8). The spindled cells had indistinct margins, and contained a scant amount of eosinophilic cytoplasm. Nuclei were elongated to ovoid and frequently hyperchromatic. Anisokaryosis and anisocytosis were moderate. Mitoses were less than one per ten high power fields. Multiple foci of mild lymphocytic to plasmacytic infiltration were also present. The mucosa overlaying the mass was partially ulcerated. The surrounding viable mucosa was composed of a hyperplastic hyperkeratotic pluristratified squamous epithelium with prominent rete ridges deepening into the submucosa, while the ulcerated portion presented a thick band of degenerating neutrophils admixed with lymphocytes and cellular debris.

**Figure 9:**
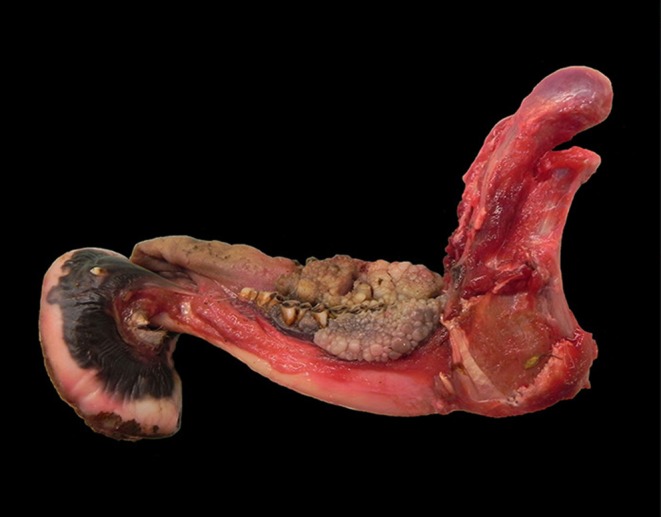
Mandible, roe deer (*Capreolus capreolus*); cauliflower-like proliferations expanding from the gingival surface and rimming the lower premolars and molars.

Additionally to the mandibular mass, multiple soft cauliflower-like proliferations (from ca. 3.5 × 2 × 1 cm to 7.5 × 4 × 2 cm) were present over the gingival surface, rimming the lateral and lingual aspects of the upper and lower premolars and molars and extending caudally up to the proximal tract of the pharynx (Figure 9). The tumor masses were shiny and light tan on cut surface.

**Figure 10:**
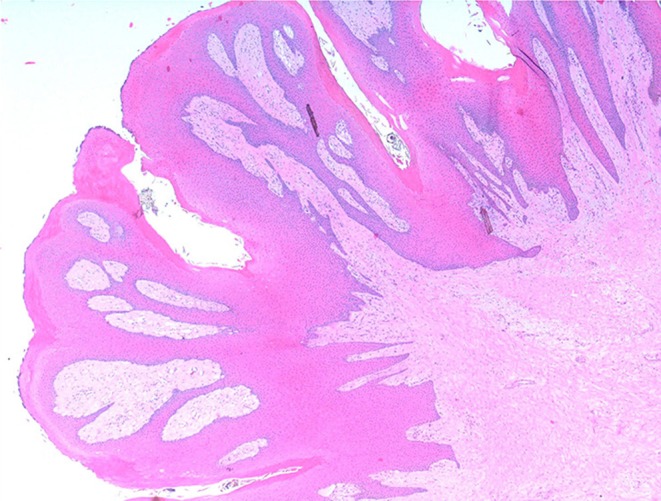
Gingival mass, roe deer; cross section of the papillary projection; H&E stain.

Histologically, the soft oral masses were arranged in papillary elements composed of keratinized pluristratified squamous epithelium with long rete ridges extending into a rich underlying fibrovascular supportive stroma (Figure 10). The epithelial neoplastic cells were polygonal to cuboidal, had distinct cell borders and moderate to large amount of eosinophilic cytoplasm. The nuclei were round to oval with stippled chromatin and contained one variably distinct nucleolus. Anisokaryosis and anisocytosis were moderate. Mitoses were less than one per ten high power fields. In between the neoplastic cells, the intercellular bridges, separated by clear spaces, were relatively prominent (spongiosis). Multiple clear intracytoplasmic vacuoles, occasionally filled with amphophilic material and pushing the nuclei peripherally, were seen within the *stratum basale* and *stratum spinosum*, while larger vacuoles, containing eosinophilic material, were seen in the *stratum spinosum* only. The upper portion of the underlying submucosal layer was poorly cellular and was composed of loose collagen fibers separated by variably extensive clear spaces, while in the deeper portion thicker fibrous bundles, more densely arranged, were seen. Multifocally, mononuclear inflammatory infiltrates composed mainly by lymphocytes and plasma cells were also observed.

### Molecular and Immunohistochemical Investigation

Given that papillomas in wild ruminants have been frequently associated with papillomaviruses (PV), the cauliflower oral masses observed in the submitted roe deer were tested for the presence of Roe deer PV DNA and antigen through PCR and immunohistochemistry, respectively. Briefly, total DNA was extracted from a section of fresh tissue excised from the oral masses using the DNeasy extraction kit (Quiagen, Hombrechtikon, CH) according to the instructions of the manufacturer. A PCR reaction was performed with a pair of PCR primers (Pap1f and Pap1r) designed for the detection of cervid PV DNA and a second PCR with broader detection spectrum capable of detecting all Delta PVs was carried out using the primers UPV1 and UPV2 (12, 15). In addition, sections of paraffin-embedded tissue of the oral masses were mounted on Xylene treated slides and processed by immunohistochemistry for the detection of PV antigen as described earlier (15). Briefly, the immunohistochemical reaction was performed using rabbit antibodies B0580 (DAKO, Glostrup, Denmark) raised against BPV1 L1 capsid protein and the reaction was visualized with a horseradish peroxidase-labeled polymer system (EnVisionTM+ Kit; Dako, Glostrup, Denmark). Cutaneous papillomas were used as control. Both PCR and immunohistochemistry yielded negative results, suggesting the absence of both Delta papillomavirus DNA and antigen in the examined lesion.

## Discussion

The gross, histological and radiological features of the mandibular mass along with its anatomic localization are consistent with an ossifying fibroma. The cauliflower oral masses were diagnosed as papillomas.

Ossifying fibroma is a slow-growing, benign, expansile fibro-osseous tumor that most commonly develops within the rostral mandible of animals (16). Its histological features are intermediate between those typical of an osteoma and of fibrous dysplasia, respectively. Morphologically it resembles a fibroma where bone forms by osseous metaplasia. The proliferative element consists of spindle cells undergoing transformation to osteoblasts producing spicules of developing bone (17). The cancellous bone is not associated with periosteum and there is a sudden transition between the fibroblastic stroma and the bony trabeculae. The latter are better developed than the bone and osteoid produced in osteosarcomas and are bordered by a layer of osteoblasts lacking the classic perpendicular arrangement to the surface of the spicules seen in osteomas. Ossifying fibromas lack cartilage formation and do not metastasize. Other characteristics include low cellularity, low pleomorphism and low mitotic index. Ossifying fibromas most frequently affect the mandible of young horses (1) but they have also been described in the lower and upper jaw of other species (2–7, 18, 19). Relatively unusual locations including the tibia (20, 21) and os penis (22) have also been reported. Other infrequent locations include that of a white-tailed deer (*Odecoileus virginianus*) affected by a debilitating ossifying fibroma of the ear (23). The ear lesion was suggested to be associated with ear tagging and with a possible virus transmission occurring during the procedure. Diagnosis of ossifying fibromas can be challenging especially when the examined lesions share significant morphological features with similar but distinct fibrous-osseous neoformations. In the case presented here, the location associated with the local invasiveness assessed through the CT scan, the absence of periosteum, the morphology and arrangements of the osteoblasts and the sharp demarcation between the fibrous and osseous components are strongly suggestive of an ossifying fibroma.

Papillomas are benign tumors usually occurring within the skin of many domestic animals but have also been described within the oral cavity, especially in dogs (24), and within the esophagus and forestomachs of cattle (25). The distinction between papilloma and fibropapilloma can be difficult especially when the papilloma is in a regressing phase. While papillomas consist of hyperkeratotic epidermal or mucosal exuberant exophytic masses supported by a fine fibrovascular stroma, fibropapillomas consist predominantly in fibroblastic neoproliferations overlaid by a hyperplastic, hyperkeratotic epidermis/mucosa (16). In this case report, although the fibrous stroma of some of the masses was relatively prominent, there were no clear groups of proliferating fibroblasts, consistent with papillomas. Cutaneous papillomas and fibropapillomas have been reported in several cervids species. These skin tumors have been repeatedly associated with species-specific PV, although this association might be more complex than previously considered (14). Genomes of deer- (White-tailed deer, *Odocoileus virginianus*) (26), Reindeer- (*Rangifer tarandus*) (10), European Elk- (*Alces alces*) (27), Roe deer- (*Capreolus capreolus*) (15), and Red deer- (*Cervus elaphus*) *papillomavirus* (12) have been sequenced and they have all been demonstrated to be member of the *Delta-papillomavirus* genus. Differently, *Bovine papillomavirus type* 4 of cattle (usually causing lesion within the esophagus and forestomach) has been determined to be a *Xipapillomavirus* (25). The PCR amplification of Delta PV DNA from a tissue sample of the oral papillomas and the immunohistochemistry directed to the detection of PV antigen were negative. Additionally, the cytopathic changes known to occur secondary to the presence of the virus, such as nuclear size reduction, perinuclear clearing (koilocytosis) and increased amounts of amphophilic foamy cytoplasm, were not obviously observed within the mucosa of this roe deer. These results suggest the absence of papillomavirus association in the observed oral masses. Alternatively, the putative presence of a PV might not have been detected because of a significant divergence with that of the best characterized PVs of wild ruminants or for the presence of an amount of viral genomic DNA under the limit of detection of the adopted protocol.

## Data Availability Statement

All the data collected during this investigation are reported in the present article.

## Author Contributions

SZ-G and FO performed the study and wrote the manuscript. T-DR performed the computed tomography examination, carried out the image analysis and interpretation and contributed to the manuscript. RP contributed to the interpretation of the bone lesions, to the diagnosis and to the manuscript. KE performed the molecular and the immunohistochemical analyses, their interpretation and contributed to the manuscript.

### Conflict of Interest

The authors declare that the research was conducted in the absence of any commercial or financial relationships that could be construed as a potential conflict of interest.
